# Treatment of chronically depressed patients: A multisite randomized controlled trial testing the effectiveness of 'Cognitive Behavioral Analysis System of Psychotherapy' (CBASP) for chronic depressions versus usual secondary care

**DOI:** 10.1186/1471-244X-8-18

**Published:** 2008-03-25

**Authors:** Jenneke E Wiersma, Digna JF van Schaik, Patricia van Oppen, James P McCullough, Robert A Schoevers, Jack J Dekker, Marc BJ Blom, Kristel Maas, Johannes H Smit, Brenda WJH Penninx, Aartjan TF Beekman

**Affiliations:** 1 Department of Psychiatry and Institute for Research in Extramural Medicine, VU University Medical Center and Academic Outpatient Clinic for Affective Disorders, Stichting GGZBuitenamstel-de Geestgronden, Amsterdam, The Netherlands; 2 Department of Psychology, Virginia Commonwealth University, Richmond, USA; 3 Department of Psychiatry, Mentrum, Amsterdam, The Netherlands; 4 Department of Psychiatry, PsyQ, The Hague, The Netherlands

## Abstract

**Background:**

'Cognitive Behavioral Analysis System of Psychotherapy' (CBASP) is a form of psychotherapy specifically developed for patients with chronic depression. In a study in the U.S., remarkable favorable effects of CBASP have been demonstrated. However, no other studies have as yet replicated these findings and CBASP has not been tested outside the United States. This protocol describes a randomized controlled trial on the effectiveness of CBASP in the Netherlands.

**Methods/Design:**

The purpose of the present paper is to report the study protocol of a multisite randomized controlled trial testing the effectiveness of 'Cognitive Behavioral Analysis System of Psychotherapy' (CBASP) for chronic depression in the Netherlands. In this study, CBASP in combination with medication, will be tested versus usual secondary care in combination with medication. The aim is to recruit 160 patients from three mental health care organizations. Depressive symptoms will be assessed at baseline, after 8 weeks, 16 weeks, 32 weeks and 52 weeks, using the 28-item Inventory for Depressive Symptomatology (IDS). Effect modification by co morbid anxiety, alcohol consumption, general and social functioning and working alliance will be tested. GEE analyses of covariance, controlling for baseline value and center will be used to estimate the overall treatment effectiveness (difference in IDS score) at post-treatment and follow up. The primary analysis will be by 'intention to treat' using double sided tests. An economic analysis will compare the two groups in terms of mean costs and cost-effectiveness from a societal perspective.

**Discussion:**

The study will provide an answer to the question whether the favorable effects of CBASP can be replicated outside the US.

**Trial Registration:**

The Dutch Cochrane Center, NTR1090.

## Background

According to the World Health Organization, Major Depressive Disorder (MDD) is the second largest healthcare problem worldwide in terms of limitations caused by illness [1]. MDD causes high rates of subjective suffering, absenteeism and utilization of medical services [2]. Both in the Netherlands and elsewhere it was found that 20% of depressions still persist after two years [3,4]. Chronic MDD causes a greater illness burden, more suicide attempts and more hospital admissions compared to acute MDD [5,6]. In outpatient mental health populations, chronic MDD affects an estimated 25–35% of depressive patients [7,8]. Unfortunately, the treatment results for this group of depressed patients are often disappointing and the existing treatment protocols are insufficiently tailored to chronic MDD [9]. For this reason, an effective psychotherapeutic treatment will constitute a welcome addition to the range of treatments currently available for chronically depressed patients.

Although there is a reasonably large body of literature evaluating cognitive as well as interpersonal therapy for nonchronic or mixed depressed samples, few studies have specifically investigated the group of chronically depressed patients [10]. A study that did target this group of patients examined the effectiveness of 'Cognitive Behavioral Analysis System of Psychotherapy' (CBASP), a form of psychotherapy that was specifically developed for chronic depression. CBASP's core procedure is called "situational analysis", a highly structured technique that teaches chronically depressed patients to fix their problematic interpersonal encounters. It is designed to teach patients that their behavior has consequences (called perceived functionality) by focusing on situational consequences to change their thoughts and behavior. CBASP conceptualizes that the interpersonal difficulties these patients encounter outside the therapy are also likely to emerge in the therapeutic relationship [11]. These difficulties are anticipated through "transference hypotheses" formulated early in treatment, which helps the therapist to recognize and resolve therapeutic impasses [9,12].

In a multisite trial involving 681 patients suffering from chronic depression, Keller and colleagues [13] compared the efficacy of an antidepressant (nefazodone), CBASP alone and their combination. After 12 weeks of treatment CBASP alone was equally effective as nefazodone alone [response rate of 48%]. The combination of nefazodone and CBASP was the most effective treatment [response rate of 73%]. After four months continuation treatment the authors found no difference between nefazodone and CBASP and found an ongoing advantage for combined treatment. Furthermore, over 85% of responders after 12 weeks of treatment maintained that response in continuation [14]. After 52 weeks of treatment, it was also found that CBASP was effective as a maintenance treatment for chronic depression [15].

In addition, it was found that CBASP was a good alternative for patients who were not motivated for, or refractory to medical treatment. For the non-responders in the study it was found that the switch from nefazodone to CBASP and vice versa resulted in clinically and statistically significant improvements [16,17].

As a result of these findings, CBASP is mentioned as an evidence based treatment option for chronic depression in the Dutch treatment guidelines [18]. However, the results of the American study have not yet been replicated. In addition, the dissemination of CBASP outside the US is limited. In order to test whether these findings can be replicated in a Dutch population we designed a randomized controlled trial on the effectiveness of CBASP. Our research questions are:

1. Is CBASP more effective and cost-effective than usual secondary care?

2. Which patient related factors predict a beneficial response?

## Methods/Design

### Study design

This study is a multi-center randomized controlled trial in which the effects of CBASP will be evaluated. CBASP, in combination with antidepressant treatment, will be tested versus usual secondary care in combination with antidepressant treatment over 52 weeks follow-up. The aim is to establish the clinical and cost effectiveness of CBASP plus antidepressant treatment in secondary care.

### Recruitment/Settings and locations

Patients will be recruited within the mood disorder departments of three organizations for mental health care in Amsterdam and The Hague. All newly registered patients will first be seen by a psychologist or psychiatrist for an intake and screening interview, which is part of a standard procedure. After the intake procedure patients' diagnosis and indication will be discussed in a staff meeting. At each site, the research-coordinator of the study will attend the staff meetings to select the patients diagnosed with chronic depression. These patients will be approached for participation in the study. The coordinator will be screening the patients on the inclusion-exclusion criteria of this study and inform them about the study. If patients are willing and eligible to participate, written informed consent will be elicited. The inclusion period will cover one year.

### Participants

#### Inclusion criteria

Patients (aged 18–65) are eligible to participate if their main diagnosis is a chronic form of depression according the DSM-IV criteria: a) a chronic major depressive disorder (i.e. existing for longer than 2 years) or b) a major depressive disorder superimposed on a dysthymic disorder or c) a recurrent major depressive disorder which, in the past 2 years, never fully remitted between the episodes. The Mini International Neuropsychiatric Interview plus (M.I.N.I. plus), a structured diagnostic interview developed in 1990 by psychiatrists and clinicians in the United States and Europe for DSM-IV and ICD-10 psychiatric disorders, will be used to assess chronic depression [19,20]. In addition, the level of symptom severity should be moderate to severe, expressed as a score of 22 or more on the 28-item Inventory for Depressive Symptomatology (IDS) [21,22].

#### Exclusion criteria

Patients are excluded from the study if: a) they suffer from one (or more) of the following disorders: a psychotic disorder, bipolar disorder, organic brain syndrome, substance dependence, or a severe borderline -, schizotypical -, or antisocial personality disorder or b) they have high risk of suicide c) they do not have sufficient command of the Dutch language necessary to participate in the study.

#### Randomization

After signing the informed consent form, patients will enroll in the study and start with the baseline interview. After completing the baseline interview, patients will be assigned at random to either the intervention group or the control group. The intervention group will receive CBASP, while the control group will receive treatment as usual. Randomization will be performed by an external researcher using a computerized random number generator. Randomization is stratified per location.

#### Assessments

Data are collected at five points in time: at baseline (T0), after 8 weeks of treatment (T1), after 16 weeks of treatment (T2), after 32 weeks of treatment (T3), and at the endpoint, after 52 weeks of treatment (T4). The Quick Inventory of Depressive Symptomatology self-report [23] will be assessed monthly. Table 1 summarizes the measures that are used at each point. The assessments will be performed by an experienced research nursing staff. At baseline and endpoint there will be a face-to-face interview besides the written questionnaires; the three assessments in between consist of written questionnaires only. The research nursing staff from the three participating organizations will participate in a two-day training course to make sure the measures are conducted the same way at the different locations.

**Table 1:** Summary of measures

**Measure**	**Baseline**	**8 week follow-up**	**16 week follow-up**	**32 week follow-up**	**52 week follow-up**
**Interview:**					
Mini plus measures diagnostics	x				x
Demographics	x				x
CBS questionnaire measures physical health	x				x
LIFE-CHART measures duration of depression	x				
NEMESIS questionnaire of childhood trauma	x				
Brugha questionnaire of recent life events	x				x
**Self report measurements:**					
Inventory of Depressive Symptoms (IDS)	x	x	x	x	x

Quick Inventory of Depressive Symptoms (QIDS)	Monthly assessment				

Beck's Anxiety Inventory (BAI)	x	x	x	x	x
AUDIT measure of alcohol consumption	x	x	x	x	x
Working alliance questionnaire-patient version		x	x	x	
Mastery scale measures locus of control	x	x	x	x	x
Social activity/participation	x	x	x	x	x
WHODAS	x	x	x	x	x
Loneliness & Affiliation Scale	x				x
NEO-Five Factor Inventory	x				x
Mood Disorder Questionnaire	x				x
Psychological Well Being (PWB)	x				x
Lack of Psychological Mindedness (LPM)	x				x
Remembered Parenting Scale (RRP)	x				
**Questionnaires for economic evaluation:**					
Registration of usage medication	x				x
TIC-P measures illness and work	x				x
Perceived need for care	x				x
TIC-P work + health care utalization self report			x	x	

**Questionnaires filled in by therapists:**	**Baseline**	**8 week follow-up**	**16 week follow-up**	**32 week follow-up**	**52 week follow-up**

Clinical Global Impression-Improvement (CGI)		x	x	x	x
Working alliance questionnaire, therapist version		x	x	x	

### Outcome measures

#### Primary outcome measure

The primary outcome measure is the reduction of depressive symptoms measured with the 28-item version of Inventory of Depressive Symptoms (IDS). The IDS is a measure of symptom severity in depression that has been used to assess acute and longer-term outcomes and has highly acceptable psychometric properties (Cronbach's alpha = .92) [21,22]. The IDS self-report version will be administered at baseline, after 8, 16 and 32 weeks and at the end of the study, at 52 weeks follow-up. Patients with a 50% symptom reduction on the IDS, are responders [13]. Remission is defined as an IDS score of 13 or less.

#### Secondary measures of outcome/predictors of response

To assess chronic depression and co morbid disorders at baseline and at the end of the study patients will receive a diagnostic interview using the M.I.N.I. plus [19,20].

Information on demographic factors (age, gender, marital status, education, professional occupation) will be collected in the baseline interview and at the end of the study.

Putative modifiers of effect are well known risk-factors for chronic depression [24], these are: level of functioning, which will be measured using the WHODAS [25], social contacts and social participation using the Loneliness & Affiliation Scale [26] and the Social Participation Scale [27], locus of control using the Mastery Scale [28] and psychological well being using the Psychological Well Being Scales (PWB) [29]. Physical health and diseases will be measured by a validated Dutch questionnaire [30], psychological mindedness using the Lack of Psychological Mindedness (LPM) [31] and parental relationship using the Remembered Parenting Scale (RRP) [31]. The Mood Disorder Questionnaire (MDQ) [32] will be used to measure bipolar symptoms, number of depressive episodes in the past five years using the Life Chart [33], anxiety using the Beck Anxiety Inventory (BAI) [34], alcohol dependence using the Alcohol Use Disorders Identification Test (AUDIT) [35], recent trauma using the Brugha questionnaire [36] and childhood trauma using the Nemesis questionnaire [37]. Personality will be measured using the NEO-Five Factor Inventory (NEO-FFI) [38] and working alliance using the Working Alliance questionnaire [39].

All measures of outcomes are widely used internationally, are reliable and valid, also for usage in the Netherlands.

#### Interventions

For this project 'the founder' of CBASP, J.P. McCullough Jr., came to Amsterdam to train the 25 therapists participating in this study. A four day workshop was given during which the basic techniques of CBASP were taught. Five therapists, subsequently, followed an intensive training by McCullough in the US to become CBASP Supervisors. They will supervise the participating therapists during the study. The therapy will be carried out as specified in the CBASP protocol [40-42]. The treatment protocol for the therapists and manual for the patients have been translated into Dutch. All therapists have to treat two patients under supervision before being allowed to treat patients in the study. All CBASP sessions will be audio taped, and the supervisors will review the audiotapes to assess the psychotherapists' adherence to the treatment procedures (McCullough, Jr. J.P. (1995). Adherence Scale. Unpublished Scale. Richmond, VN: Virginia Commonwealth University). Supervision will be given on a weekly basis.

##### CBASP

The central idea of CBASP is that chronically depressed patients fail to recognize the connection between their behavior and interpersonal/environmental consequences. In other words, they do not grasp their own contribution to the life difficulties they encounter. The major goals of CBASP are to help patients 1) to understand the consequences of their behavior, 2) to change their patterns of coping, and 3) to improve their interpersonal skills [9].

CBASP is highly structured, focuses on teaching social problem skills, and makes use of regular homework assignments. The main focus is on interpersonal problems with significant others, but also in the therapist-patient relationship. The relationship with the therapist is used as a tool to help patients to become more aware of their impact on others and to distinguish between adaptive and maladaptive relationships [9].

CBASP consists of a total of 26–30 sessions in a period of a year. CBASP starts with bi-weekly sessions in the first 4 weeks followed, in principle, by one session per week from week 5–16. If establishing the therapeutic relationship proves particularly difficult, the two-weekly sessions can be prolonged for four more weeks. After 16 weeks (16–20 sessions), 4 sessions will be given once every two weeks during weeks 17–25, and as a maintenance treatment there will be 6 monthly sessions of CBASP during weeks 26–52.

##### Treatment as usual

At the three sites, treatment as usual is based on the existing Dutch multidisciplinary guidelines for depression [18]. This means that, besides optimal medical care, treatment as usual can consists of evidence-based psychotherapies, such as Cognitive Behavioral Therapy, Interpersonal Psychotherapy, or Short Psychoanalytic Supportive Psychotherapy [43]. Other interventions that may be part of the treatment offered for chronically depressed patients are supportive or structured activities and treatments that focus on relaxation, assertiveness, running, or other tasks. The treatment package received by patients in the treatment-as-usual condition will be registered throughout the study.

##### Pharmacotherapy

In both conditions drug treatment will be given, consisting of guideline' driven antidepressant medication [18]. The pharmacotherapy will be supported by 'clinical management' [44]: brief sessions in which patients will be informed about the importance of adherence and the effects and side effects of medication. The psychiatrist will change the dose or medication when necessary. The use of medication (name, dose given, and blood levels if available) will be registered for all patients participating in the study. Patients who refuse to use medication will not be excluded, because CBASP has also been found to be effective as a monotherapy for chronically depressed patients [13].

#### Economic evaluation

An economic evaluation will be performed from a societal public health perspective. All direct and indirect costs will be taken into account. Both direct costs within the health care system (e.g., costs of visits to GP, specialist, and therapist, hospital admissions, and medication use) and direct costs outside the health care system (e.g., costs of self medication, consultations with complementary medicine, and informal care) will be measured and valued. All direct costs will be considered because it is difficult to discern which costs are associated with the depressive disorder and which not. The cost-price of the CBASP intervention will be assessed using the bottom-up method. Data on health care utilization and work absenteeism are collected by the Perceived Need for Care questionnaire (PNCQ) [45] and the Trimbos/iMTA questionnaire for Costs associated with Psychiatric Illness (TIC-P) [46], which patients will fill out four times during the study, at baseline, after 16 weeks, after 32 weeks and at the end of the study, after 52 weeks. Medicine utilization will be registered at baseline and during the study until the end. Costs of health care utilization will be calculated using cost prices, if available. Otherwise tariffs will be used. Indirect costs will be calculated using the friction cost method.

#### Sample size

Based on the findings of the study of Keller and colleagues [13] it is estimated that the addition of CBASP to the existing treatment will lead to 25% more response in the intervention condition compared to the control condition. To demonstrate this difference 60 patients in each condition (beta 0.80 and alpha 0.05) are necessary [47]. Assuming a drop-out percentage of 25%, this means that 160 participants need to be recruited.

#### Analysis

Analysis of covariance, controlling for baseline value will be used to estimate the overall treatment effectiveness (difference in IDS score) at follow-up. The primary analysis will be by 'intention to treat' using double sided tests. Further exploratory analyses will also assess the impact of other explanatory factors and will model the time course of effects using the 16-week and 32-week measurements in a panel analysis. Sensitivity analyses will include estimating 'on treatment' effects, CACE (complier average causal effects), and imputation of any missing values. More sophisticated techniques will be used, like GEE analysis, to measure the longitudinal effects.

The main aim of the economic analysis is to compare the two groups in terms of mean costs and cost-effectiveness. Analysis will be performed according to intention-to-treat principle. The primary outcome measure of the study will be included in the cost-effectiveness ratios. Cost-effectiveness ratios and cost-utility ratios will be calculated using bootstrapping techniques according to the bias corrected percentile method. Bootstrapped cost-effect pairs will be graphically represented on a cost-effectiveness plane. Sensitivity analyses will be performed to study variations in prices or resource uses that are uncertain and significantly contribute to the total costs.

#### Ethical principles

The participation in the study is voluntary. Participants are informed that they can cancel their participation at any time without disclosing reasons for their cancellation and without negative consequences for their future care. Participants will sign an informed consent.

#### Vote of the ethics committee

The design and conduct of the study was approved by the Medical Ethics Committee of the VU University Medical Center, Amsterdam.

## Discussion

This study protocol is presented here to offer researchers the opportunity to consider the methodological quality of this study with a critical view. Therapists can benefit by considering the information regarding the practical applications of the proposed protocol on chronically depressed patients in secondary care.

The number of studies examining the effects of psychotherapy on chronically depressed patients is very small, while chronic depression affects a large group of patients in secondary care and this subgroup is notable for poorer subjective well being and impaired social and occupational functioning compared to non-chronically depressed patients [10]. Research into the effectiveness of CBASP can make an important contribution to the improvement of care for chronically depressed patients. According to the results of Keller et al. (2000), 73% of these patients can be effectively treated if CBASP is combined with antidepressant medication. CBASP can also be an alternative for patients who are not motivated for or refractory to pharmacological treatment. Furthermore, CBASP could also be effective as a maintenance treatment [13-17].

### Strengths and limitations

Many methodological requirements for a high quality trial are met. Allocation is concealed through randomization by an external researcher. Recruitment of the patients will be done after the standard intake procedure at each site. As this study takes place within the mood disorder departments of three organizations for mental health care in the Netherlands the results can, to a large extent, be generalized to the population of chronically depressed patients seen in secondary care in the Netherlands.

A limitation of the present study is that the assessments will be performed by research nursing staff who cannot be kept blind to the treatment condition of the included patients, as is always the case in trials studying the effects of psychotherapy. Nor are the patients blind to treatment condition. In this study, however, the main outcome measures are self-report questionnaires, which means that they will not be influenced by the research nursing staff.

### Timeframe of the study

In October 2006 a pilot study, supervised by McCullough, has started to enable the therapists to gain experience with this innovative intervention. In May 2007 the randomized treatment study has been started up. Month 1–12: Screening and inclusion of chronically depressed patients at the three locations. Start of the data collection for the treatment study. Supervision of the treatments. Start of data entry and purging of databases. Month 13–24: Completion of data collection. Completion of purging and analysis of the data. Publication on these short-term findings.

Month 24–36: Completion of data collection of the follow-up. Publication on the effectiveness of CBASP and the follow-up effects. Dissemination of the results (e.g. presentation of study results at national and international conferences). Working on the implementation of CBASP in the Netherlands.

### Description of risks

There are no specific risks related to this study.

## Competing interests

The author(s) declare that they have no competing interests.

## Authors' contributions

PvO, DvS and AB developed the design of the randomized clinical trial and participated in writing the article. JM, RS, JD, MB, KM, BP and JS advised on the content of the article and supervise the project. JW is the principal investigator and writer of this manuscript. All authors have read and approved the final version of the manuscript.

**Figure 1:**
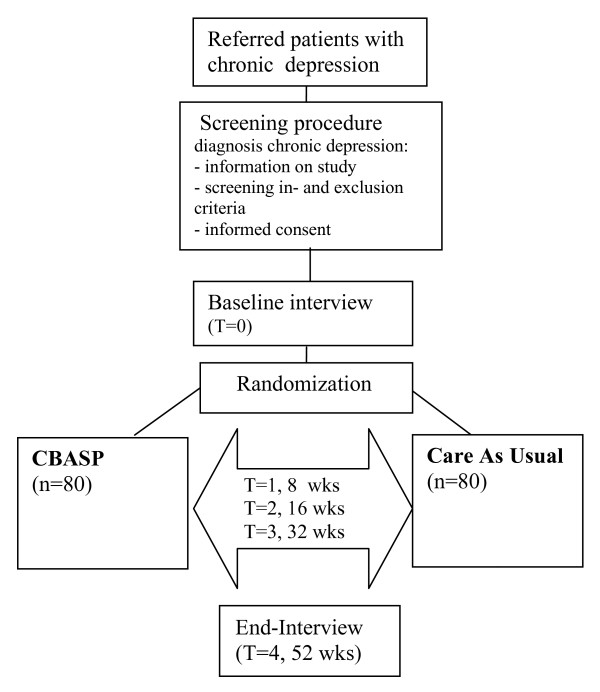
shows the procedures in a flow chart.

## Pre-publication history

The pre-publication history for this paper can be accessed here:


